# Causation of Fever

**Published:** 1867-11

**Authors:** 


### EDITORIAL AND MISCELLANEOUS.

---

### CAUSATION OF FEVER.

Malarial Fever, its cause and nature, are themes upon which much time and labor have been expended; and now, discussions are rife as at any time heretofore.

We would not be understood as entertaining the idea that no advancement has been made—no facts established—but that other mooted questions are yet to be settled.

Below, we give extracts of a discussion between the physicians of Macon, Ga., on this subject, as connected with forest growth. The question arose from the proposition to clear up and bring into cultivation some low lands—a public reserve—lying contiguous to the city, and to which is attributed the immense amount of malarial fever found amongst the citizens in a portion of the town.

We first make extracts from an argument by Dr. D. W. Hammond, before the Medical Society of Macon; and then draw upon an article from the pen of Dr. J. Emmett Blackshear, on the opposite side of the question:

"I will here frankly admit, that the first medical men of the age, and our most learned authors from time immemorial, have always maintained that it was absolutely necessary to preserve the forest growth intact, in order to secure the citizens from malaria or ague poison. Ah! this is very strange. In all places thus environed and protected in Europe and America, in fact all over the habitable globe, *chills and fevers prevail from year to year.* How is this? These tall and majestic oaks stretch out their strong arms in every direction to protect us. The jungle constructs a thick network near the surface of the earth for the same purpose, yet Malaria creeps through the interstices, and our defenceless women and children, yea, our strong men also, shake from head to foot with chills, in the immediate vicinity of these barriers, placed there by our forefathers for our protection. It does appear to me that this supposed barrier—this forest growth—causes rather than prevents chills and fevers. I therefore join issue with the learned sages, who hold a different opinion. I say they are all wrong. We always should judge a tree by its fruit. When we place a sentinel to guard us against marauders and enemies, if he fall asleep at his post, or is not vigilant, and thereby the enemy be admitted, what ought to be done? Why he should be removed of course. And this brings us directly to the subject under discussion.

The inhabitants in the South-eastern part of the city have chills and fever every year; and often winter and summer, notwithstanding the forest growth and jungle which have been preserved by our wiseacres to shield them. But they have not been efficient—have not been vigilant. They have slept upon their posts. Chills have crept into the city by them. Our peaceful citizens have been disturbed and distressed year by year. They are no protection, but a nuisance, and ought to be entirely eradicated.

"Mr Chairman, this is an age of progress, and every man has a right to think for himself. We should never do wrong because our ancestors did wrong. We must analyze and investigate for ourselves; must have opinions of our own, and dare to express and vindicate them. The old books with their foolish dogmas to the contrary notwithstanding. Why sir, we were taught by the old books to bleed a patient with Pneumonia and Billious Fever three times a day in some instances. They were suffered to swallow Calomel *ad libitum.* The physician that would pursue such a practice now, would be considered a humbug or a mad-man. When we were called to a man with fever, if he was vomiting bile we generally gave him a dose of Tart. Emet. to eject it faster—that is, to assist nature—the *vis medicatrix naturæ.* If he were purging off billious matter, we gave him 20 grains Calomel, and about 40 grains Jalap, in a half teacupful of molasses, to be repeated *pro re nata.* This was given to clean out the primæ viæ, and to thoroughly emulge the hæpatic apparatus. If the patient happened to die, we had the consolation of knowing that we treated the case heroically and energetically, in technical language—methodically and secundem artem. I have long since quit this foolishness. I have been rebellious for many years past.

"My father always planted his potatoes in hills. I have long since ascertained from observation and experience that they will do just as well, if not better, in ridges. I am sorry I have so little veneration for ancient opinions and notions.

"I have asserted that this forest growth and jungle instead of protecting us from fever, is really and *ipso facto* the cause of the evil; and will now proceed to the best of my feeble ability to prove it. I maintain that wherever fevers prevail, three things, or elements, must coëxist, viz: 1st, Heat; 2d, Moisture, or water; and 3d, Vegetable matters.

"These elements must exist in combination, and under particular circumstances, to generate malaria or fevers. Neither one of them, nor two combined, will eliminate malaria. Pure water when acted on by heat is harmless. Dry vegetable matters, exposed to the sun's rays, evolve no mephitic gas. Green vegetable matter, exposed to the sun, is also harmless. And green vegetable matter exposed to the sun, if immersed in water, is innoxious, provided the water is cold, or in a high latitude. But these three elements, under favorable circumstances, create a pestilential gas, or something (call it what you please) which will produce febrile diseases.

"The more clearly we investigate the subject, the more extensively we make our observation, the more certainly we shall find this doctrine rests on the strong foundation of truth. The great difficulty in this matter arises from our losing sight of the etiology of malarial fevers, and by not studying the medical topography of the locations of autumnal as well as equatorial epidemics, and the variations of the weather, and the temperature, preceding and during their presence, as well as the nature of the country surrounding or adjacent to these annual fevers.

"Wherever fever prevails to any extent, it is during the summer and autumnal months, and is more extensive during, or shortly after the rainy season; and in those places surrounded by extensive marshes, and covered by forest growth and jungle, and aquatic plants. Dr. Johnson says in Benin, new and old Calabar, fevers prevail throughout the year, and these places are surrounded by dense woodlands and marshes. All other cities similarly situated in Africa, Europe, and in America, have been the graves of unnumbered thousands of the human race. The city of Rome has been scourged with intermittent and remittent fevers for hundreds of years. And every medical man of any intelligence knows that it is surrounded by the Campania De Roma, which is thickly set with forest growth and impenetrable jungle. Dr. Johnson, who has written the ablest work ever published on tropical climates, and the diseases incident thereto, observes 'that in so luxurient a climate as Bengal, and so fertile alluvion as the Delta of the Ganges, we may well suppose that every spot—almost every particle of matter—teems with animal and vegetable life."

"The author goes on to show, that it is during this dissolution of animal and vegetable matter preparatory to new combinations and successive reproduction, that a certain inexplicable something is extricated which operates with such powerful and baleful influence on the functions of the human system. This exhalation—this malaria—is capable of concentration, or rather accumulation; for when it is detained amid woods and jungles, (the forest growth) in such localities, especially during the rainy and hot seasons, and no regular breezes dissipate it, and when the beams of the sun are obscured except at intervals by dense clouds and shade, *it*, the ague poison becomes exceedingly powerful and destructive to life. When the rains subside, and the sun pours his heating rays upon these accumulated exhalations that have been caught and detained in the leaves of the trees, and the winds rise, this poison is wafted and diffused over towns and cities, causing disease and death. One other quotation from Dr. Johnson's book, in reference to the Batavian epidemic which occurred in the year 1800. 'There are two small islands near this place, viz, Onrust and Edom; Onrust is about three miles from the mainland of Java; Edom is situated nine miles from the shore, out at sea.' A circumstance that the Doctor thought must insure its salubrity. I will now glance at the medical topography of these two islands. Onrust is a small island near the main land, (mark what he says) 'well cleared of trees, underwood and jungle. A part of this island is daily covered twice by the tides.' The English squadron were placed here when sick, as there was comparitively but few cases of intermittent fever, and those cases of a mild type (mark this).

"The island of Edom, on the other hand, nine miles from the Batavian exhalations, is covered with trees, long grass and jungle.' The surgeon of the squadron, in consequence of the soldiers being subject to chills occasionally and too of a mild type at Onrust, removed them to Edom, supposing it would be more healthy, being covered thickly with trees and timber. The Doctor goes on to remark: 'The loss of seamen I have not been able to ascertain,' but he says, 'almost the whole of the sick that were removed from Onrust, the cleared island, to Edom, the wooded island, perished, in consequence of the poisonous malaria—in a concentrated form, which was contained and issued from the trees by which they were surrounded.' I could go on and show many other instances where the crew suffered from fevers under like circumstances. And so of every other country where chills and fevers prevail, you will find that they are surrounded by, or in near proximity to the cause of the evil—forest growth and jungle —and that these epidemics prevail during the hot months and after the rainy seasons.

Having premised the foregoing remarks as a foundation, I shall proceed to apply them to the subject under consideration. Suffer me to advert to the two islands just spoken of, Onrust and Edom. Why was Onrust more salubrious than Edom? and too when situated more unfavorably for health, being only three miles from the Batavian or coast exhalations. Simply because it has been denuded of its vegetation. The forest growth had been felled and removed from time to time by seamen for wood.

"When the winds from Batavia freighted with pestilential effluvia swept over it, there were no trees heavily set with foliage *to catch and accumulate this poison.* It was dissipated in the air and absorbed by water. And in consequence of the island being cleared and to some extent cultivated, there was but little cause of disease.

"No vegetable matter to be decomposed by the heat of the sun. This island it will be remembered was covered to a great extent twice a day by the tides. Plenty of moisture and a burning sun, but no vegetable matter to be decomposed. And forsooth Onrust was delivered from the plague. Does not this apply to the South-eastern part of the city of Macon, and the forest growth in the reserve. Let us proceed a little farther. How was it with Edom? This island was father out at sea, removed further from the pestiferous shores of Batavia. Not so much irrigated by tide water as the former. But her dense forest growth caught every breath of malaria from the coast.

"Dr. J. M. Johnson, one of the editors of the Atlanta Medical and Surgical Journal, has written a very able article on the subject in the March number of that Journal. The following remarks are, in the main, quotations from that valuable communication. He says in every country where the arts and sciences have been developed and cultivated, discriptions of criptogamic plants abound showing their diffusion to be paripassu with the universe. Their poisonous properties and their peculiar seasons of growth—the minuteness of their sprout, their love of darkness and tainted soils, and heavy atmospheres, are, according to Dr. Mitchell, proverbial.

"Dr. Mitchell says, 'The mushroom (fungi) is generally distant from the plants and animals—mere fortuitous developments of vegeto-animal matter. Of all vegetables they are the most highly animatised. The criptogamic family of plants have no organs of generation and propagate their species by spores.' They embrace the litchens, mushrooms, and algæ. They vary in size, from a mere point, requiring the aid of the microscope to more than 30 pounds weight. Their increase is magical. From a single spore, on the authority of Dr. Carpenter, the physiologist, they may grow in a night to the size of a large gourd, and are estimated to contain four thousand five hundred millions of cells. The spore is the reproductive element (or palmella as it is sometimes called), and many times smaller than the cells. But both are so small as to require a microscope of great power to see them. Dr. Saulsbery has given them the name of earth miasma, from their minuteness.

"The palmella, or poisonous criptogam, is the variety to which your attention is more specially directed. *It abounds in bogs and low, damp, shady places:* its generic name is algæ—vulgarly known as green moss, and generally seen in still stagnant water (algæ confervia), etc. It is so minute as to be absorbed by the skin—taken into the stomach by the saliva—and into the lungs by respiration. There is one fact I will mention in regard to this morbific agent. It is innoxious *when wet*, but only active during the process of *dessi-*

*cation* when *dried* by the sun. In this state it is exhaled into the atmosphere—Dr. Saulsberry says it rises from 35 to 100 ft. It is exhaled at night and settles to the ground during the day. Hence the insalubrity of night air has been proverbial in all latitudes. It grows at night only; it may be seen in abundance in the morning, but it all passess away before evening —it cannot stand sunlight. It is of various colors; red, white, green and lead-color. The white which we often see on wood, grass and leather, is said to be of the palmella type. I must let this notice of the criptogramic theory suffice, and will now endeavor to show that this theory sustains my position, beyond the shadow of a doubt. These criptogams are only seen in low, damp, shady places—only rise a certain distance above the surface. That above the summit plain of the cool night exhalations these bodies do not rise, and *intermittents do not extend.* That the day air of malarial districts are quite free from these *palmalloid spores*, and the causes that produce intermittents. Wherever the palmella grows to any extent, you will find intermittent fever. Beyond the palmella line it is rarely seen.

"North of Lancaster, near a rich prairie and near an old canal, the people are universally subject to chills, and the palmella grows there luxuriantly every year. In short, in all places where the criptogams flourish, the inhabitants are subject to chills and fever."

The following extracts are from an article by Dr. J. Emmett Blackshear, of Macon, Ga., on the subject under discussion among the physicians of that city:

"But it is generally conceded that the great amount of sickness that annually prevails in the Southern and Southwestern portions of the city, during the months of summer and autumn, is caused by malaria generated in the low lands of the City Reserve. How is this evil to be remedied? I answer, not by destroying the beautiful trees planted there, for wise and benificent purposes, by the hand of the Creator. In the name of humanity I charge you not "to cut down the forest growth entirely," &c., as my worthy friend advises; for if you do, the city of Macon will be scourged with a pestilence rivaling in horror that which desolated in former times, the proud metropolis of our mother country.

"Onrust, the island devoid of vegetation, of which he speaks in connection with Edom which was 'covered with trees, long grass and jungle,' is by no means a parallel case. I admit that if the City Reserve was cleared up and placed in cultivation, the inhabitants of that portion of the city above mentioned, would *eventually* become less subject to fevers, but woe be unto them during the transition, *as proposed*, from forest to field, and for some time thereafter. When the scorching rays of the summer sun falls upon the unprotected vegetable matter that has been long accumulating upon the moist earth, it will then be seen whether or not these forest trees have been 'unfaithful sentinels.' Aye, it will be found when too late, that the proper course would have been not to execute these sentinels as traitors, but to remove every obstacle to the execution of their duty. Yes, remove the jungle, and drain the lands. This much I would earnestly advise. If the evil is not thus remedied, it will not be remedied by the destruction of those proud old trees, whose outspread branches have for ages sported with the swift-winged winds, bidding defiance to the tempest and the storm. At all events, try it first; if it fails, go no further; for rest assured that by so doing the evil will be augmented. If it succeeds, then, *in due time*, cut down if you will, these old inhabitants of the forest, as you would desert in his old age, a faithful servant who had served you long and well, simply because he could be of no further use.

"I might cite instances innumerable to prove the correctness of my position, but I deem it unnecessary, for there will be but few, I opine, who will read this article without being reminded of cases where evils, such as I would avert, have been produced by the incautious destruction of forest growth. The Doctor admits "that the first medical men of the age, and our most learned authors from time immemorial, have always maintained that it was absolutely necessary to preserve the forest growth intact, in order to secure the citizens from malaria or ague poison." If such is the opinion formed from careful observation, by the most learned men of the profession in all ages, let us not hastily abandon it for a new one, based as I earnestly believe, upon an erroneous hypothesis. Let the experiment be at least made in some locality where there are not so many lives at stake; and when tried, my word for it, it will be found, as it has in every instance on record, that the evil would be augmented rather than abated."

" The Doctor very correctly says " that when fevers prevail, three things or elements must co-exist, viz: 1st, Heat, 2d, Moisture or Water; and 3d, Vegetable matter. Neither one of them," he says, "nor two combined, will eliminate malaria." Then according to his own theory, there can be no necessity for destroying the trees. Remove the moisture by ditching and drainage, and he tells you that no malaria can be generated.

He admits "that many instances can be cited where the forest growth has been removed, especially if done in the summer months, and the neighborhood has become sickly afterwards. But you must recollect," he says, 'that this state of things only lasts for one or two seasons, and better endure this for a short time than endure it forever.'

" Now if the desired object can be accomplished without subjecting the vicinity to this awful scourge—as I maintain it can by simply draining the land—would we be excusable for running the risk, simply to put a few dollars in the city treasury?"

We regret that our space will not allow the insertion of the entire articles of the gentlemen engaged in this discussion; but as we have selected that which embodies the main arguments upon the purely scientific question, we hope no injustice will be done either party by the extracts. Both the gentlemen are our intimate, personal friends, and we labor only to promote the cause of science.

From the discussion we have not sufficient data to form an opinion as to the correctness of conclusions on the scientific question involved. It is not definitely stated whether the "forest growth" is confined to the marshy land, or, to any extent, intervenes between the bog and the city. Upon this we think rests the whole question of propriety in removing the growth.

It is known that *malaria*—whether in the form of algoid spores, it is not our purpose now to discuss—is generated in stagnant pools and marshy low-land, when they receive the washings from the hills of fertile cultivated fields, or earth upturned otherwise, either directly upon them, or through an overflowing stream laden with the product of the newly disturbed soil. The production is likely to take place whether the stagnant pools are shaded with "jungles" and "forest growth," or open to the direct rays of the sun; provided, that warm dry weather succeeds such overflow or washing, so that the stagnant water gradually subsides, leaving the algæ, if you please, exposed to the air.

This *something*, thus generated, whether of cryptogamous or other origin, rises and is wafted by the breeze in a horizontal direction one or more miles, *if no obstruction be met with*. In its onward course, however, should walls or the dense foliage of a thickly set forest growth intercept, its poisonous effects are not certainly seen beyond. Continued rains, or very frequent overflows, prevent the development of this poisonous production, so long as they continue.

